# Supplementary material for: Complexity synchronization analysis of neurophysiological data: Theory and methods
Source: Front Netw Physiol. 2025 May 14;5:1570530. doi: 10.3389/fnetp.2025.1570530 (PMC12116615; doi:10.3389/fnetp.2025.1570530)
Supplement: Supplementary file 1 [file DataSheet2.pdf]

Supplementary Material

1 SUPPLEMENTARY DATA

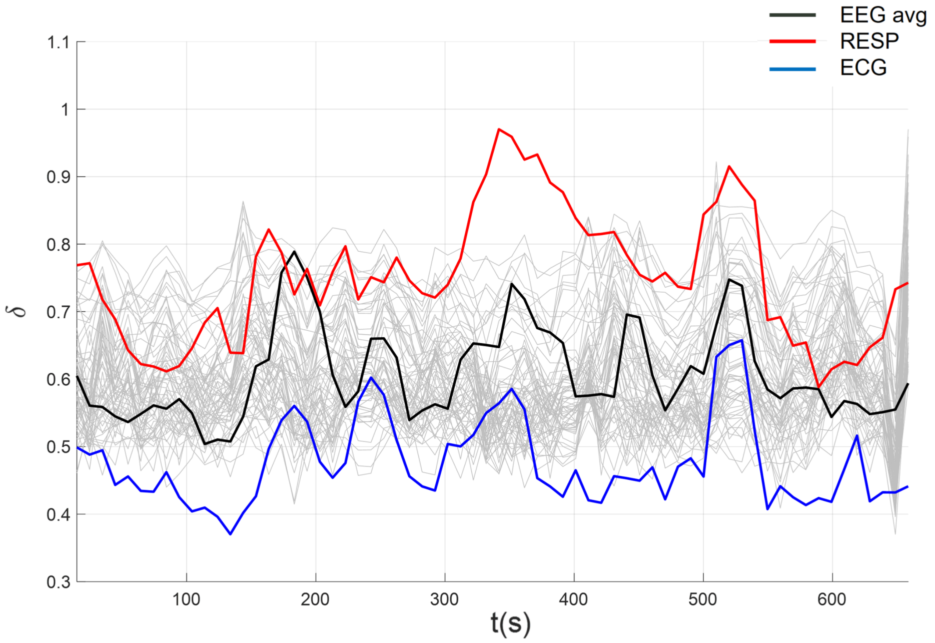

Figure S1. CS among EEG, ECG, and the envelope of the Hilbert transform of RESP using automated parameters selection (Compare with Figure 10 in body of manuscript).

	EEG	ECG	RESP
EEG	-		
ECG	.70*	-	-
RESP	.61*	.55*	-

Table S1. Correlations among scaling indices of the mean of 64 EEG channels, ECG, and the envelope of the Hilbert transform of RESP (*p< .01) (Compare with Table 4 in body of manuscript)

	EEG	ECG	RESP
EEG	-		
ECG	.87*	-	
RESP	.69*	.77*	-

Table S2. Correlations among scaling indices of the mean of 64 EEG channels, ECG, and filtered RESP for S024 Low time stress condition (*p< .05)

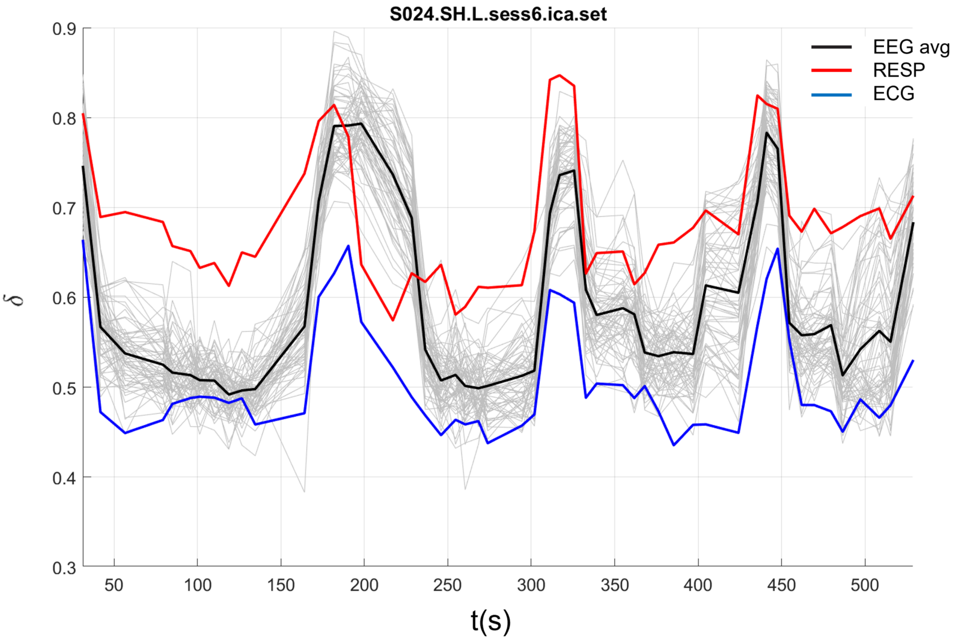

Figure S2. Significant complexity synchronization for subject S024 among EEG, ECG, and filtered RESP in Low time stress condition.

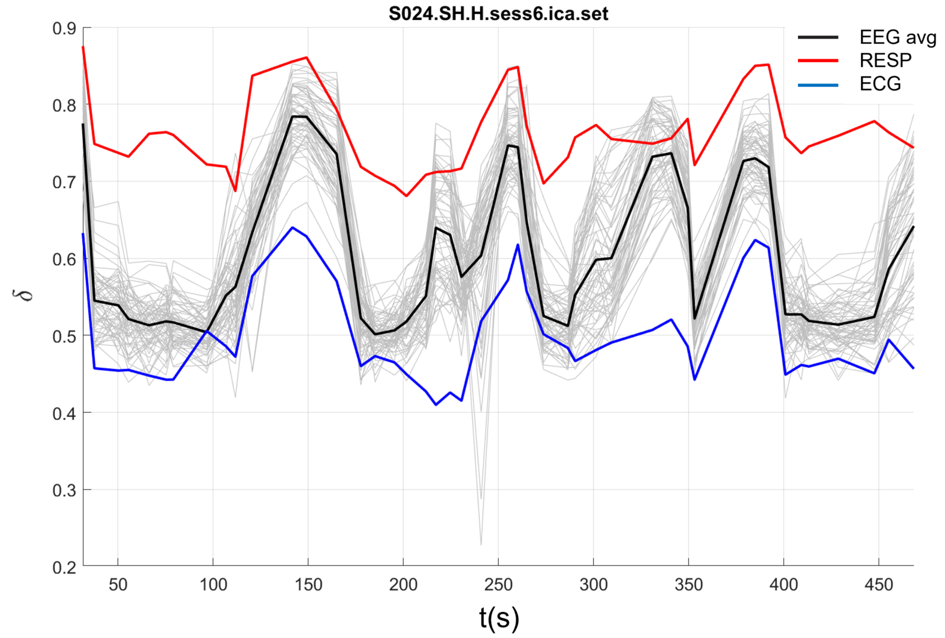

Figure S3. Significant complexity synchronization for subject S024 among EEG, ECG, and filtered RESP in High time stress condition.

	EEG	ECG	RESP
EEG	-		
ECG	.80*	-	
RESP	.77*	.86*	-

Table S3. Correlations among scaling indices of the mean of 64 EEG channels, ECG, and filtered RESP for S024 High time stress condition (*$p < .05$)

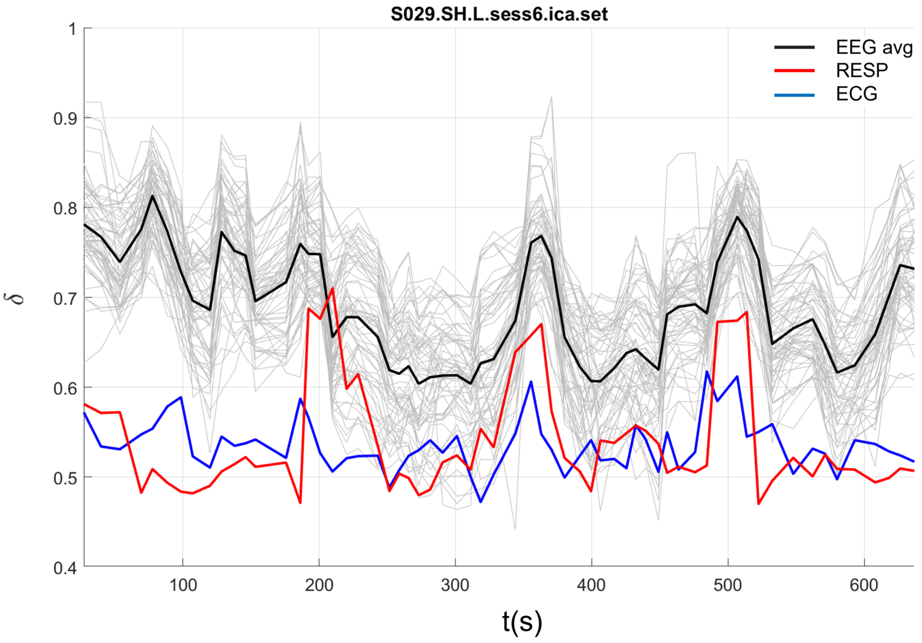

Figure S4. Non-significant complexity synchronization for subject S029 among EEG, ECG, and filtered RESP in Low time stress condition.

	EEG	ECG	RESP
EEG	-		
ECG	.54*	-	
RESP	.32*	.21	-

Table S4. Correlations among scaling indices of the mean of 64 EEG channels, ECG, and filtered RESP for S029 Low time stress condition (*p< .05)

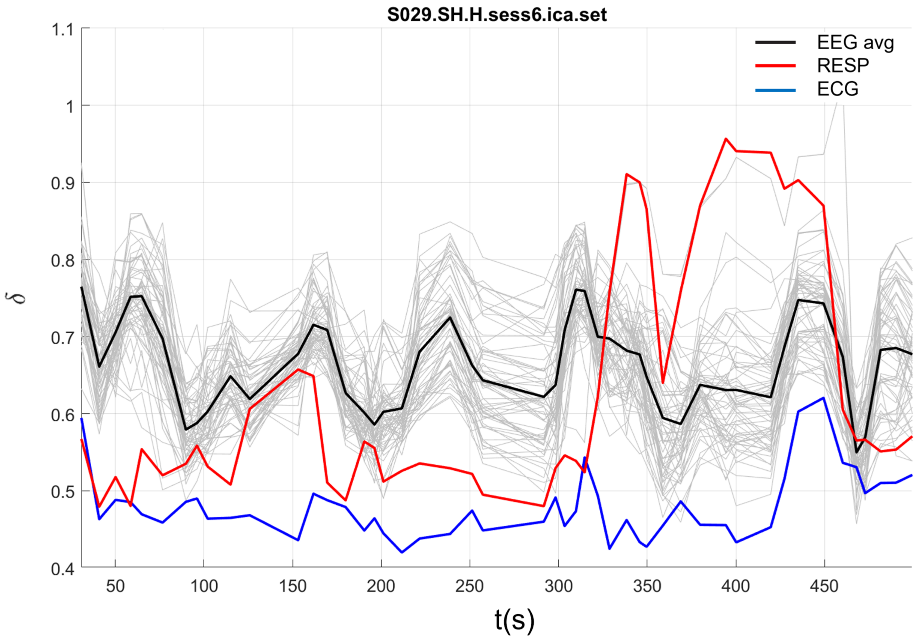

Figure S5. Non-significant complexity synchronization for subject S029 among EEG, ECG, and filtered RESP in High time stress condition.

	EEG	ECG	RESP
EEG	-		
ECG	.37*	-	
RESP	.02	.04	-

Table S5. Correlations among scaling indices of the mean of 64 EEG channels, ECG, and filtered RESP for S029 High time stress condition (*p< .05)

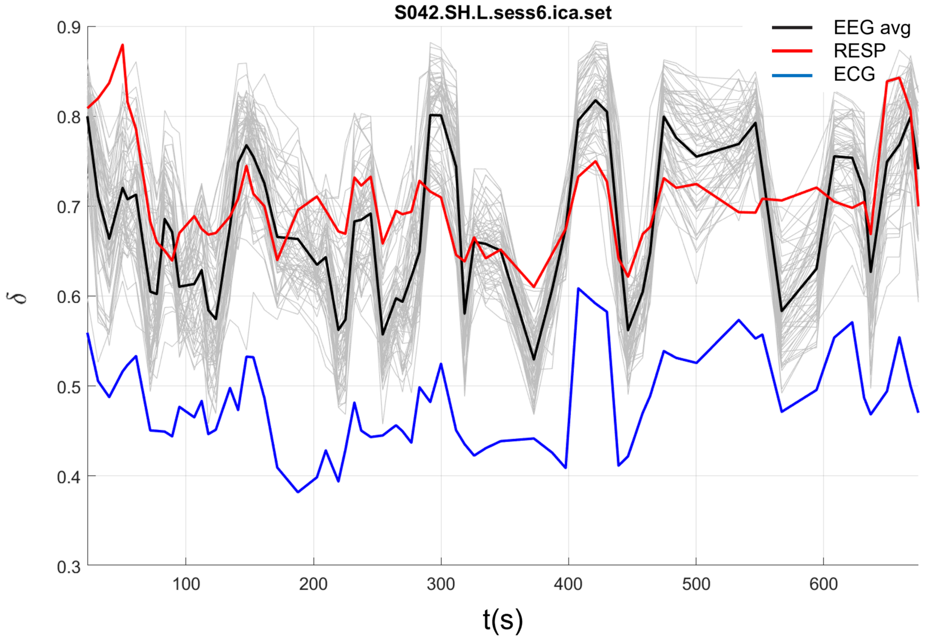

Figure S6. Significant complexity synchronization for subject S042 among EEG, ECG, and filtered RESP in Low time stress condition.

	EEG	ECG	RESP
EEG	-		
ECG	.74*	-	
RESP	.53*	.54*	-

Table S6. Correlations among scaling indices of the mean of 64 EEG channels, ECG, and filtered RESP for S042 Low time stress condition (*p< .05)

	EEG	ECG	RESP
EEG	-		
ECG	.65*	-	
RESP	.10	-.09	-

Table S7. Correlations among scaling indices of the mean of 64 EEG channels, ECG, and filtered RESP for S042 High time stress condition (*p< .05)

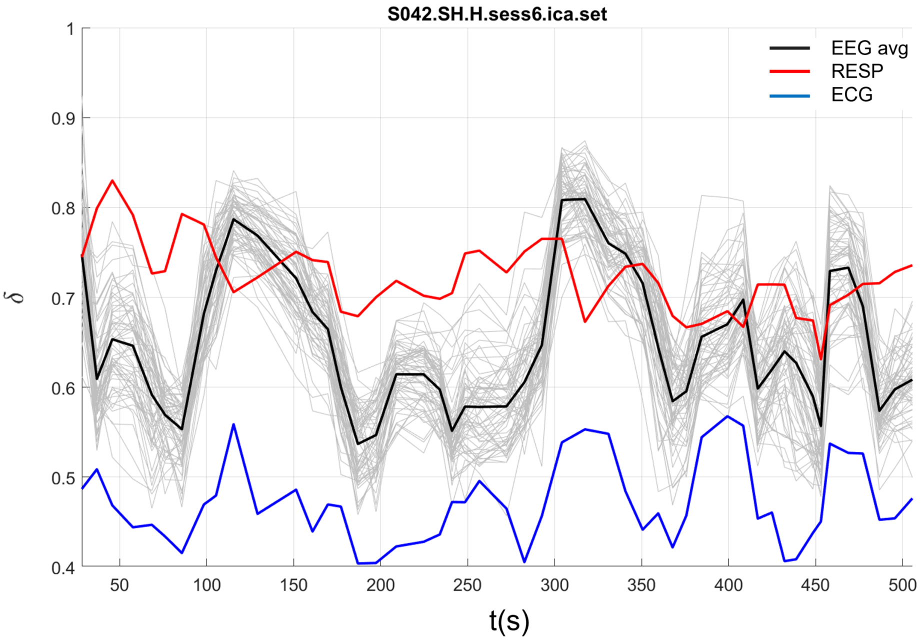

Figure S7. Non-significant complexity synchronization for subject S042 among EEG, ECG, and filtered RESP in High time stress condition.
