# Supplementary figures and images for: Complexity synchronization analysis of neurophysiological data: Theory and methods
Source: Front Netw Physiol. 2025 May 14;5:1570530. doi: 10.3389/fnetp.2025.1570530 (PMC12116615; doi:10.3389/fnetp.2025.1570530)

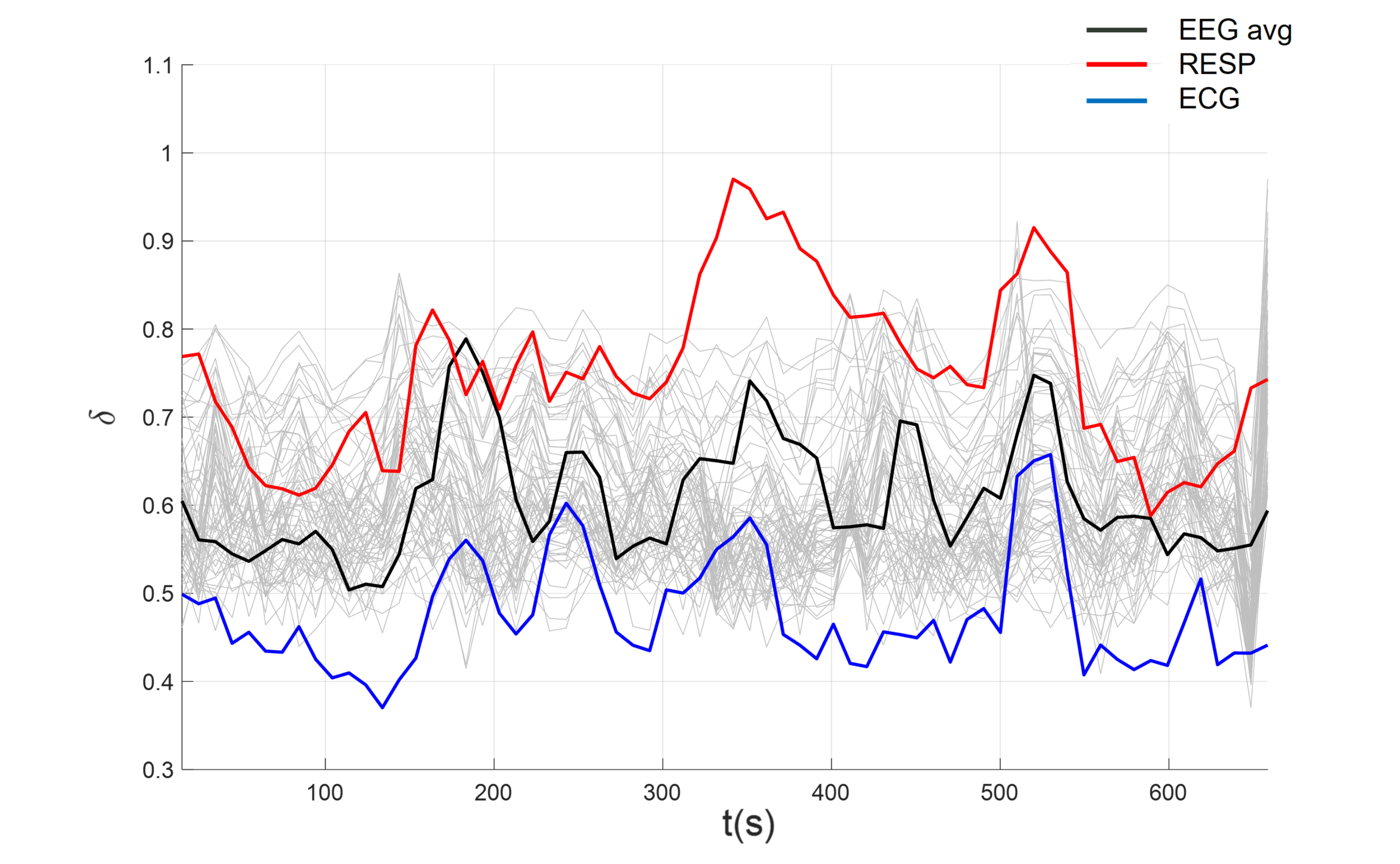

Supplement: Supplementary file 2 [file DataSheet1.zip › Figures/CS-envRESP.png]

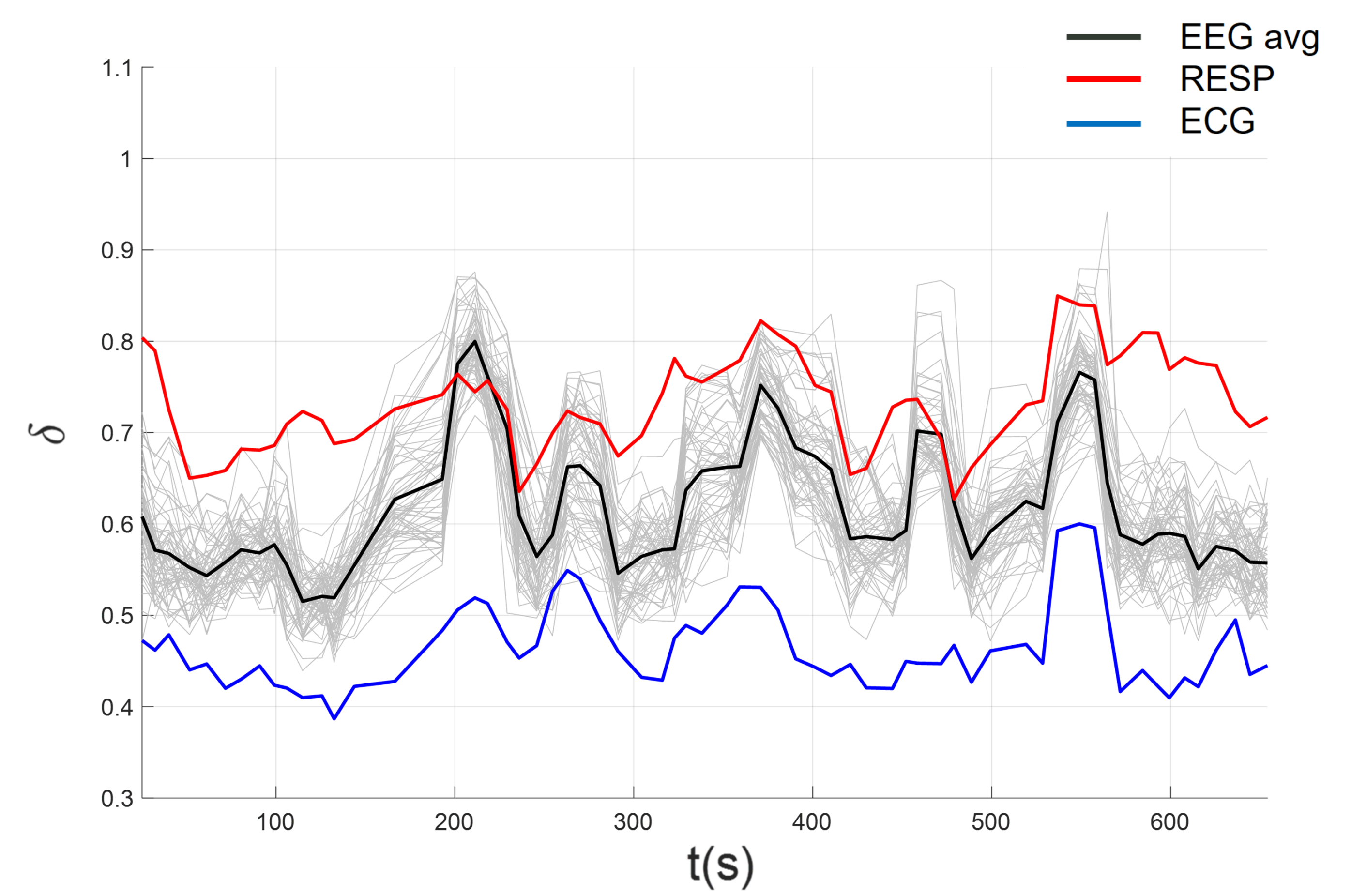

Supplement: Supplementary file 2 [file DataSheet1.zip › Figures/CS-filteredRESP.png]

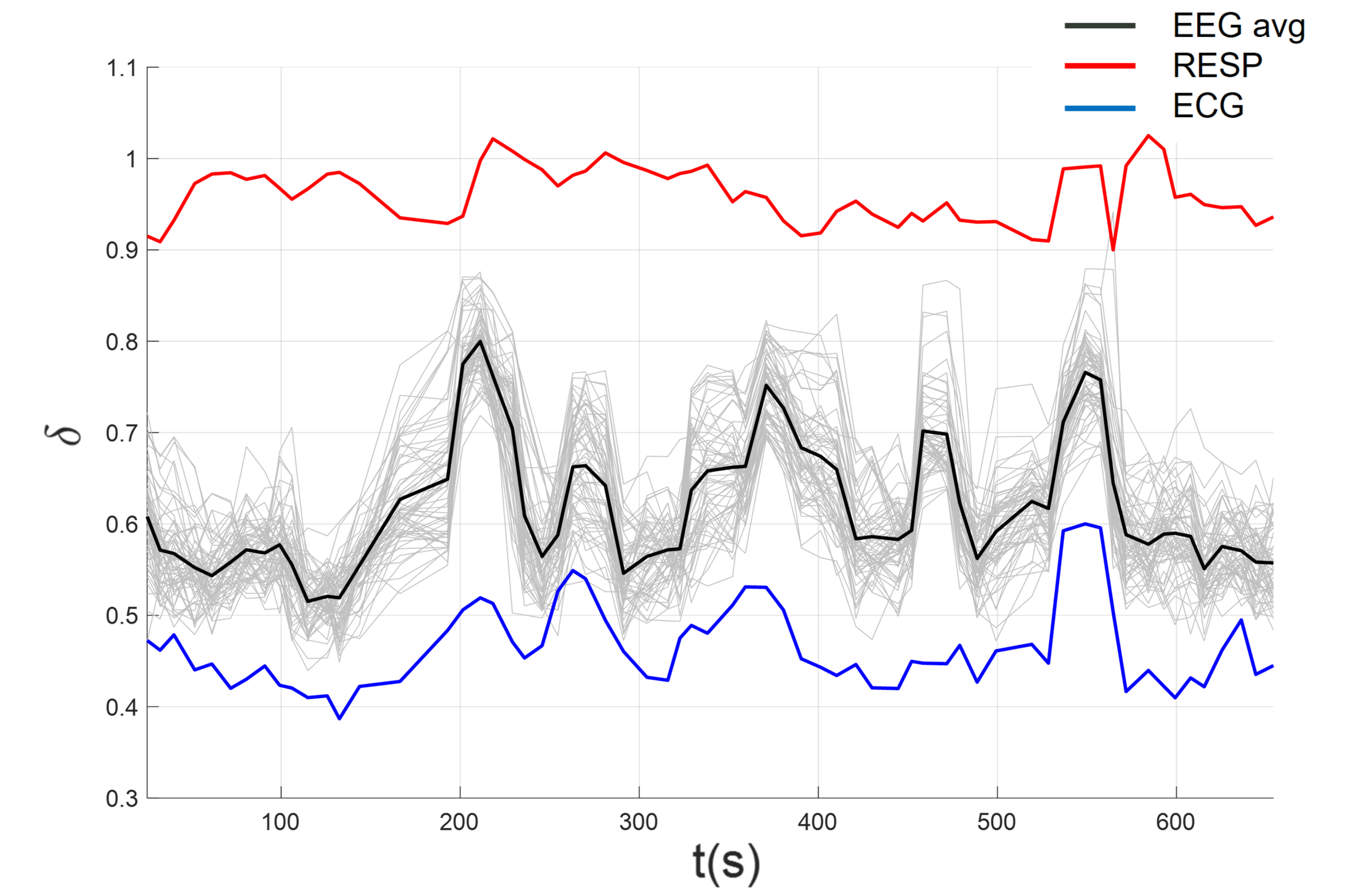

Supplement: Supplementary file 2 [file DataSheet1.zip › Figures/CS-unfilteredRESP.png]

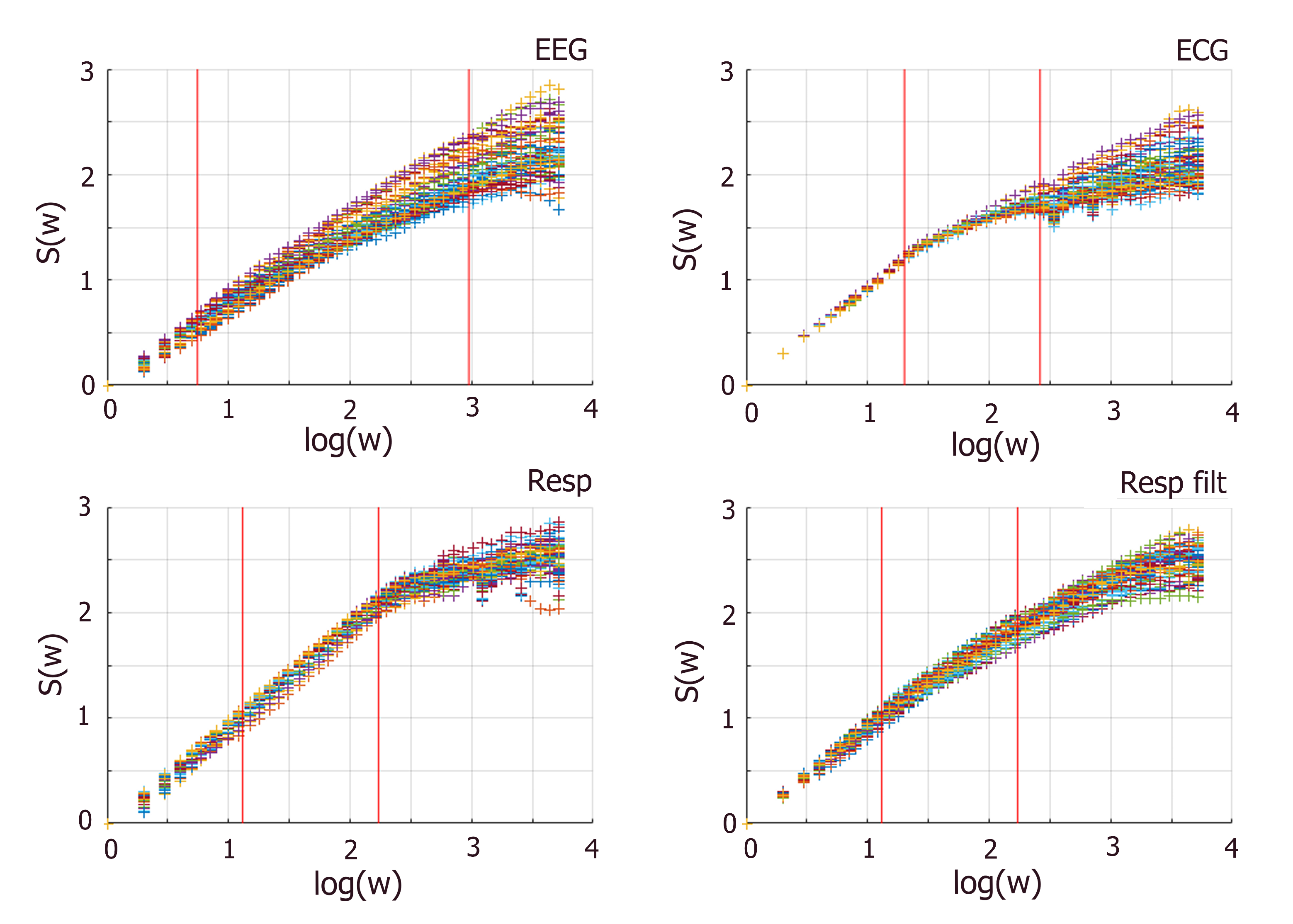

Supplement: Supplementary file 2 [file DataSheet1.zip › Figures/Entropy_RESP.png]

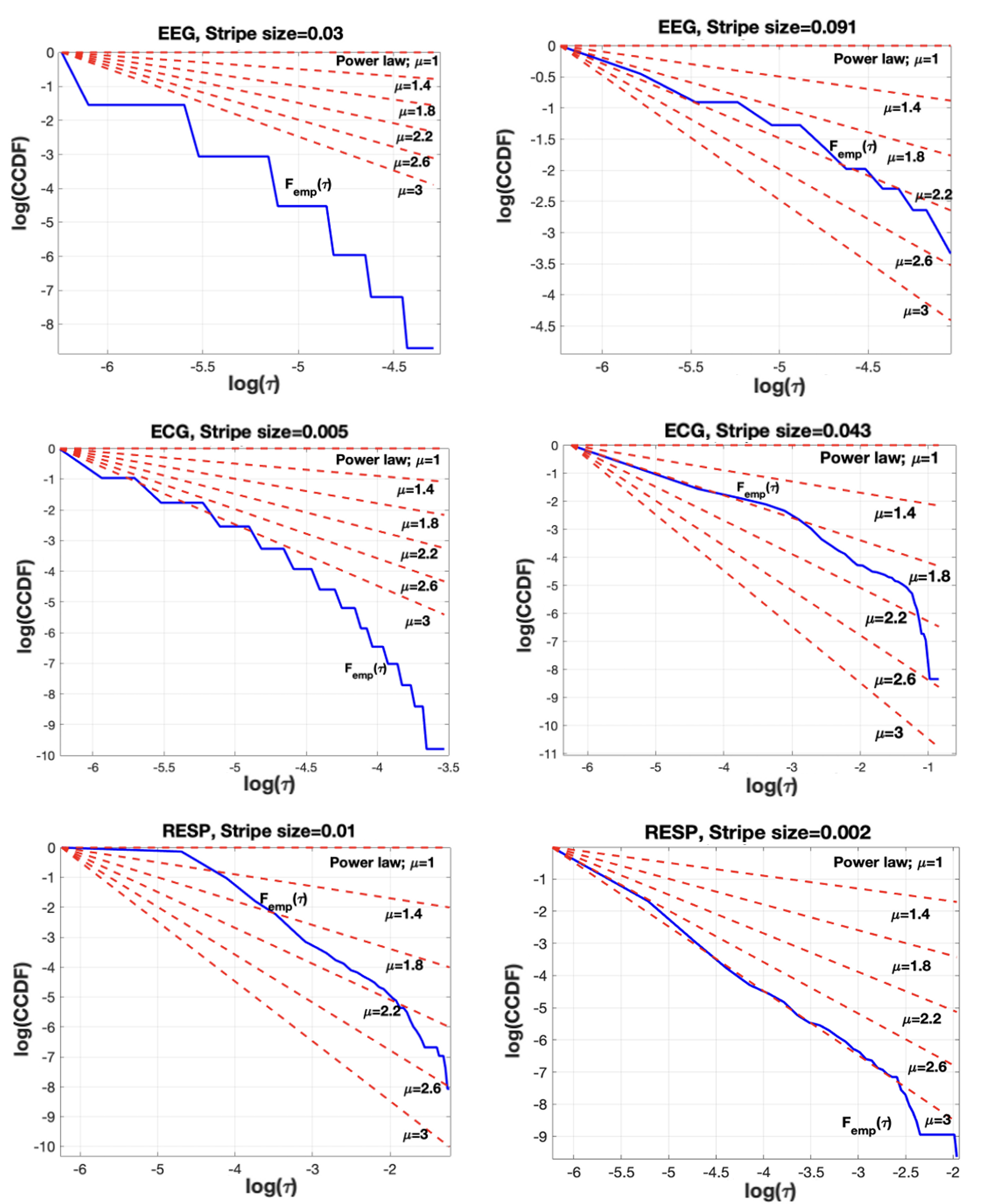

Supplement: Supplementary file 2 [file DataSheet1.zip › Figures/Fig3_new.png]

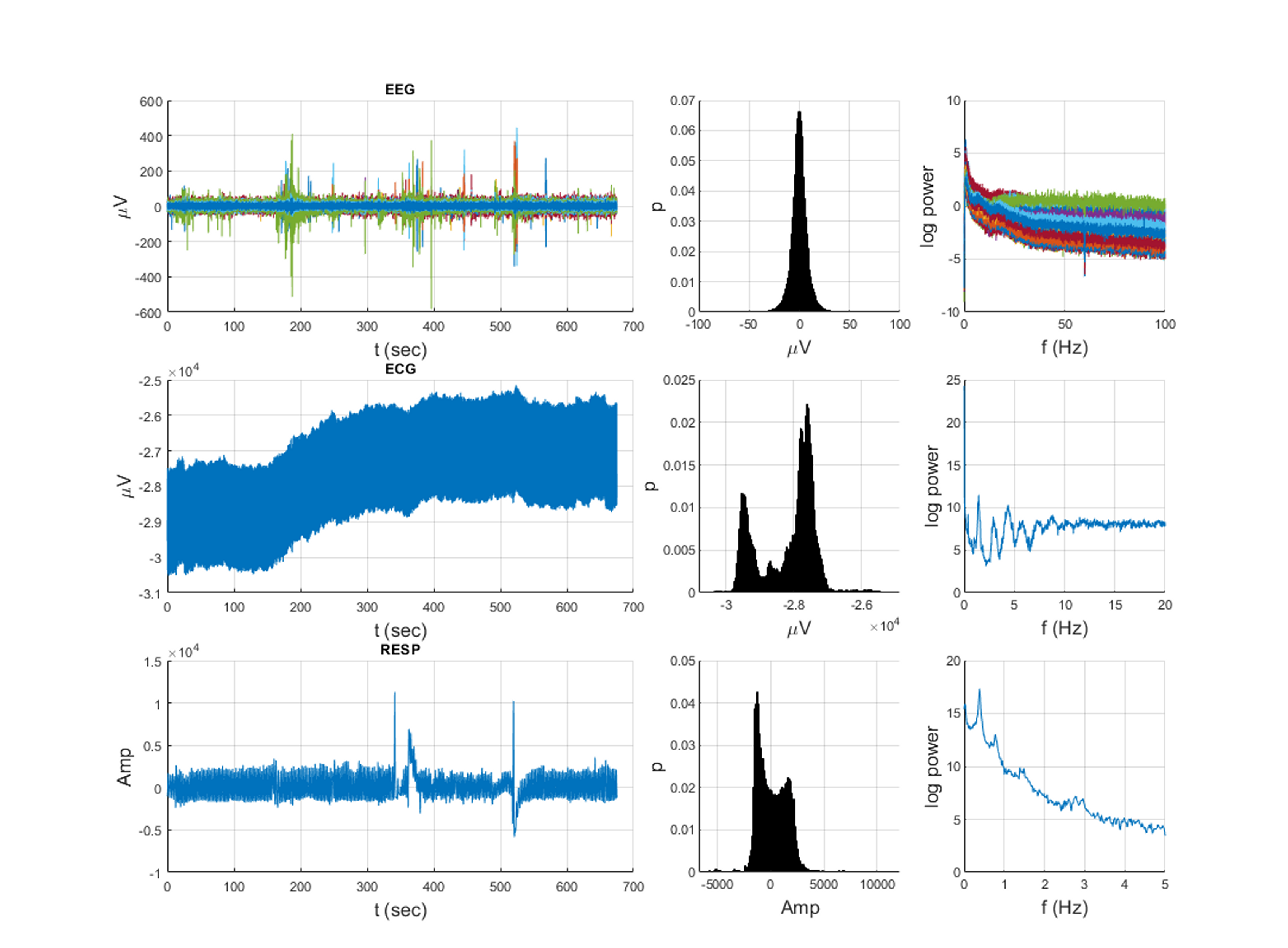

Supplement: Supplementary file 2 [file DataSheet1.zip › Figures/Figure01TimeSeriesDiversity2.png]

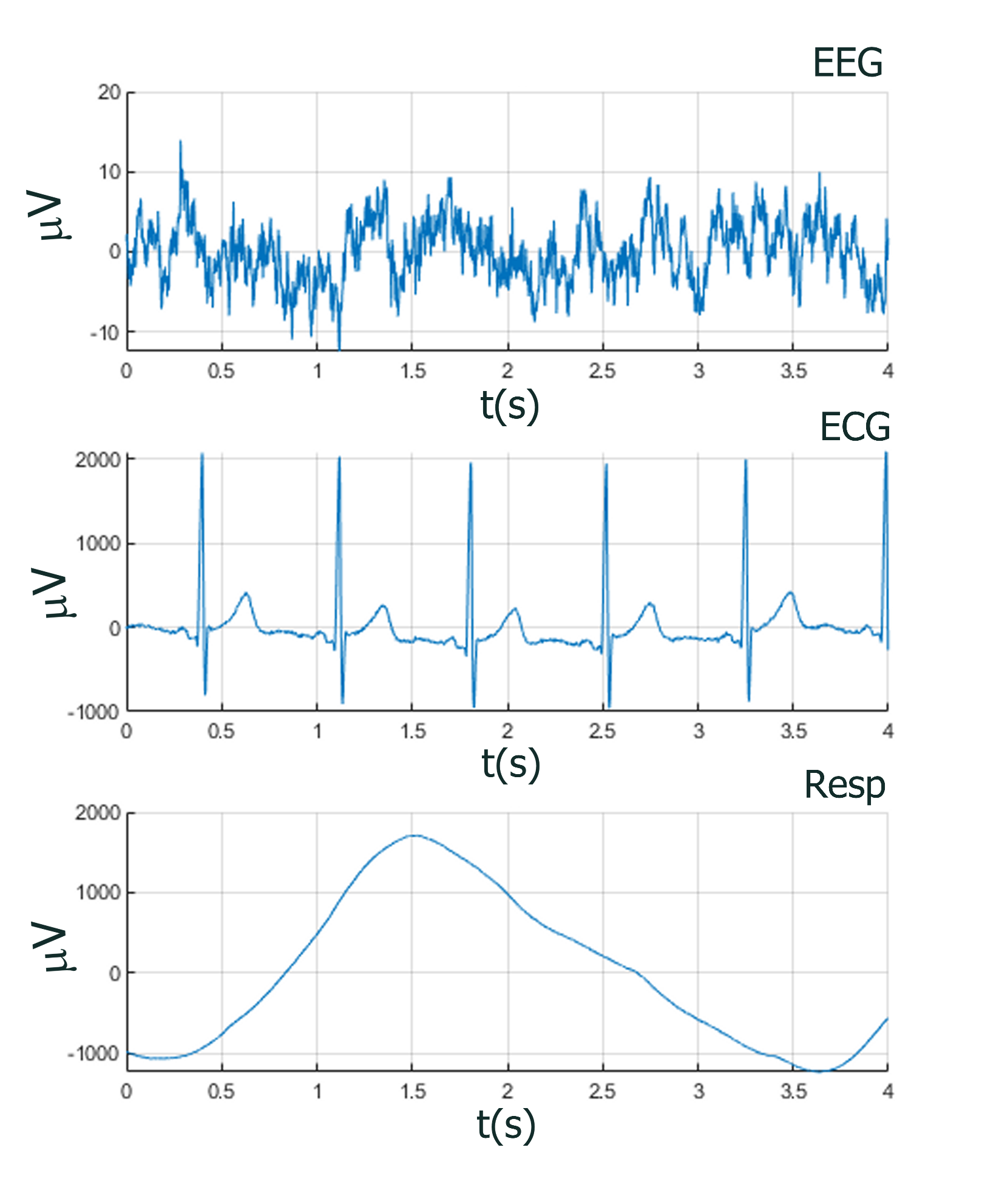

Supplement: Supplementary file 2 [file DataSheet1.zip › Figures/Figure02EEG+ECG+RESP.png]

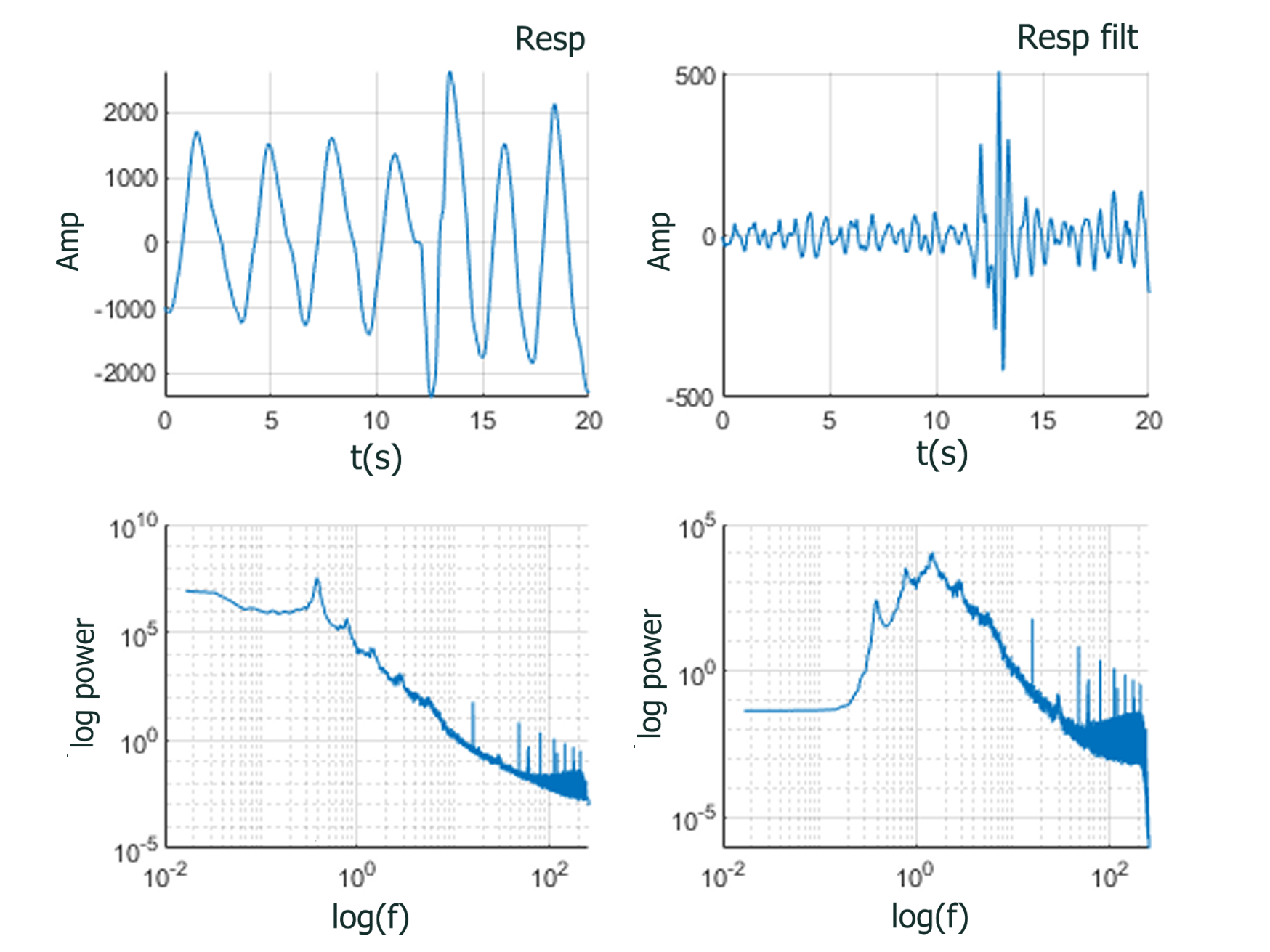

Supplement: Supplementary file 2 [file DataSheet1.zip › Figures/RESP_SPEC.png]

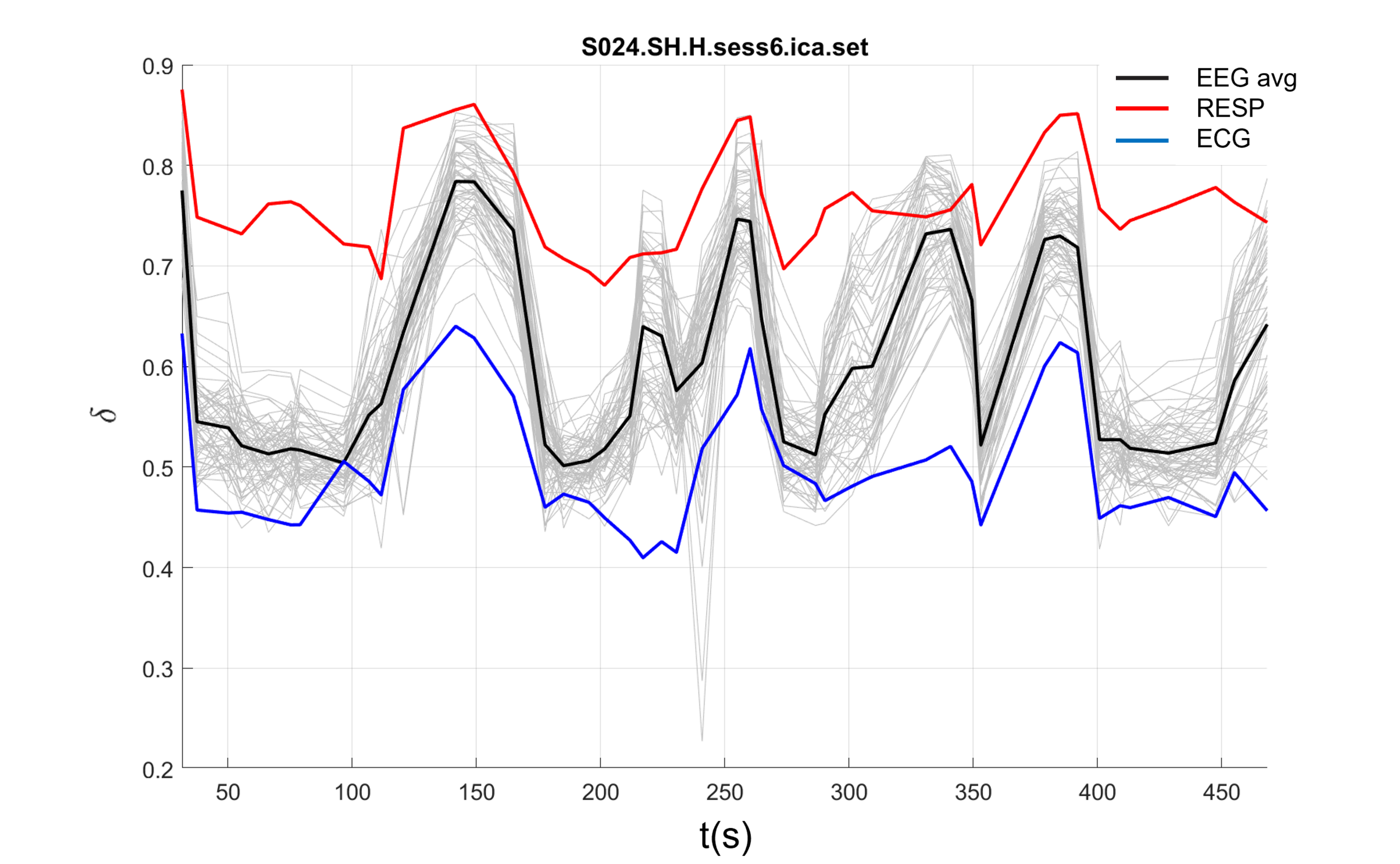

Supplement: Supplementary file 2 [file DataSheet1.zip › Figures/S024.SH.H.sess6.delta_SIG.png]

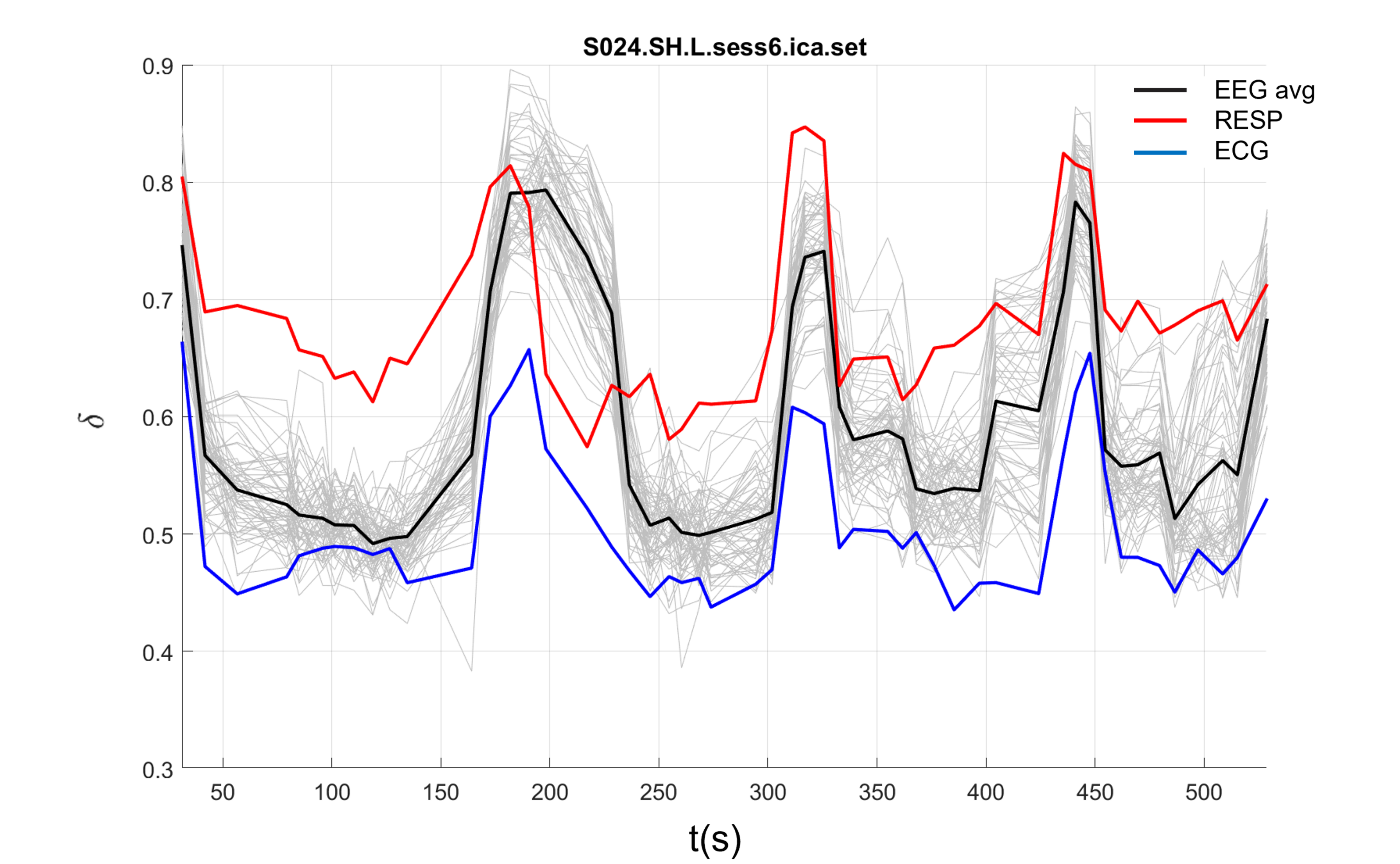

Supplement: Supplementary file 2 [file DataSheet1.zip › Figures/S024.SH.L.sess6.delta_SIG.png]

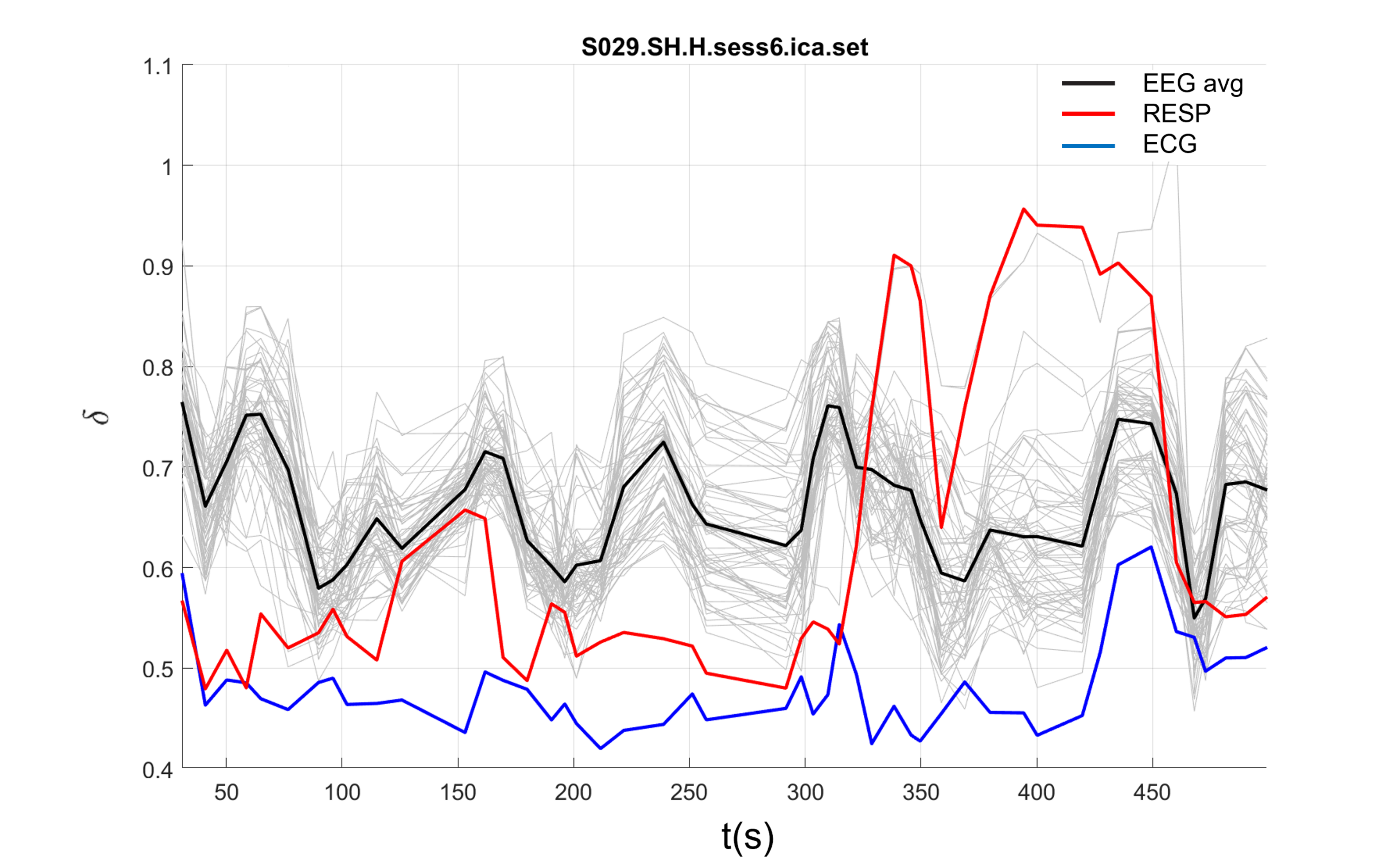

Supplement: Supplementary file 2 [file DataSheet1.zip › Figures/S029.SH.H.sess6.delta_NS.png]

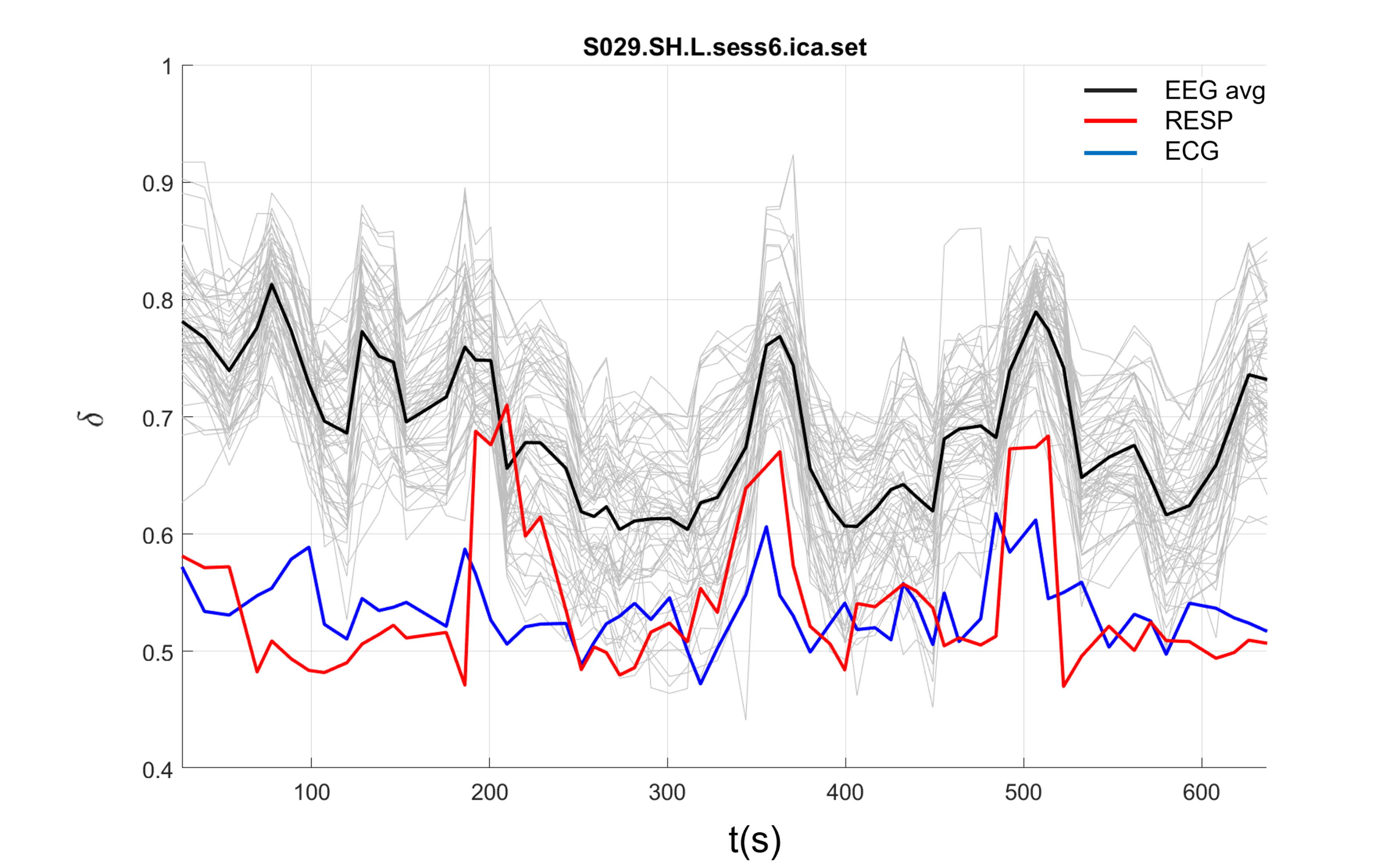

Supplement: Supplementary file 2 [file DataSheet1.zip › Figures/S029.SH.L.sess6.delta_NS.png]

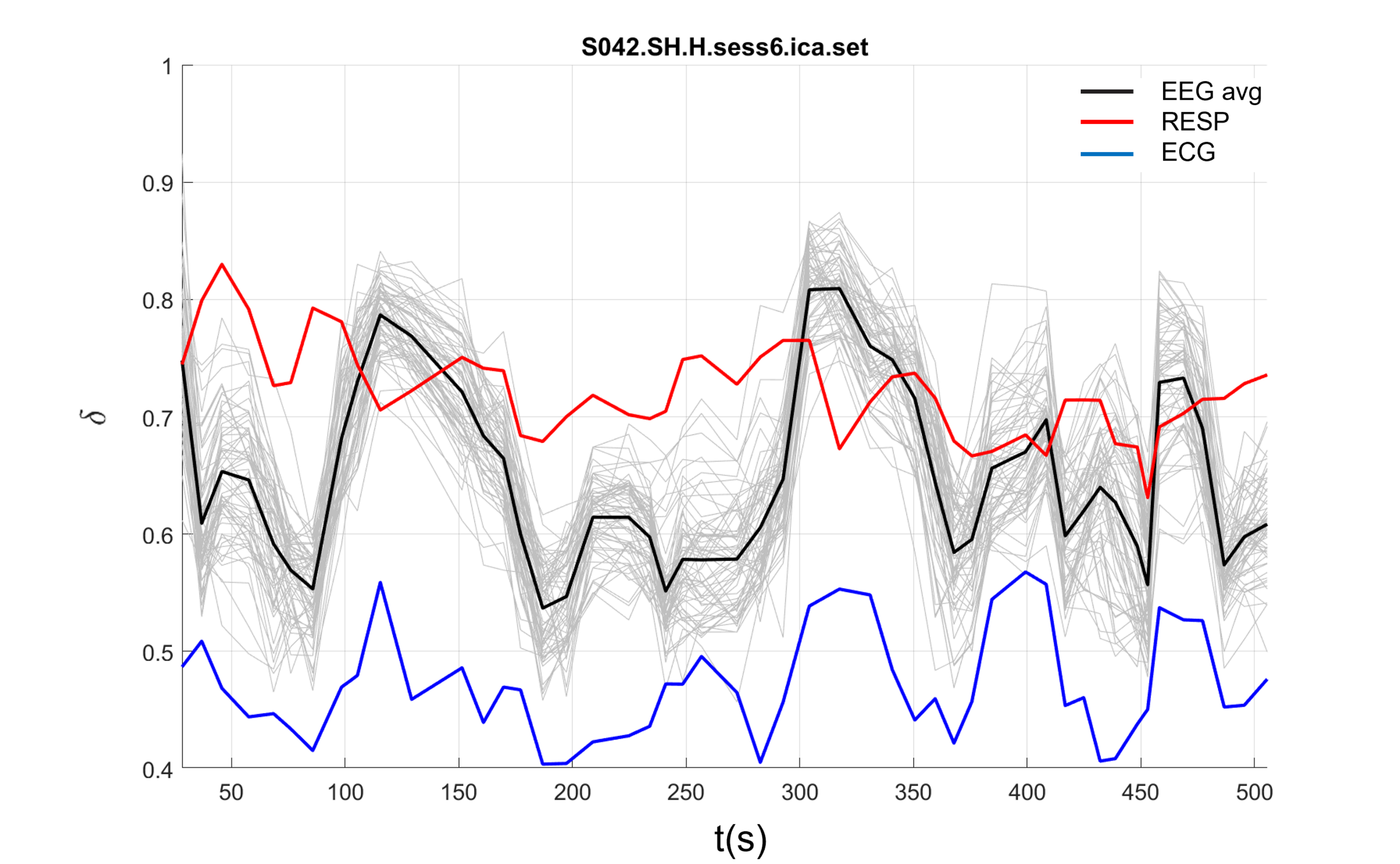

Supplement: Supplementary file 2 [file DataSheet1.zip › Figures/S042.SH.H.sess6.delta_NS.png]

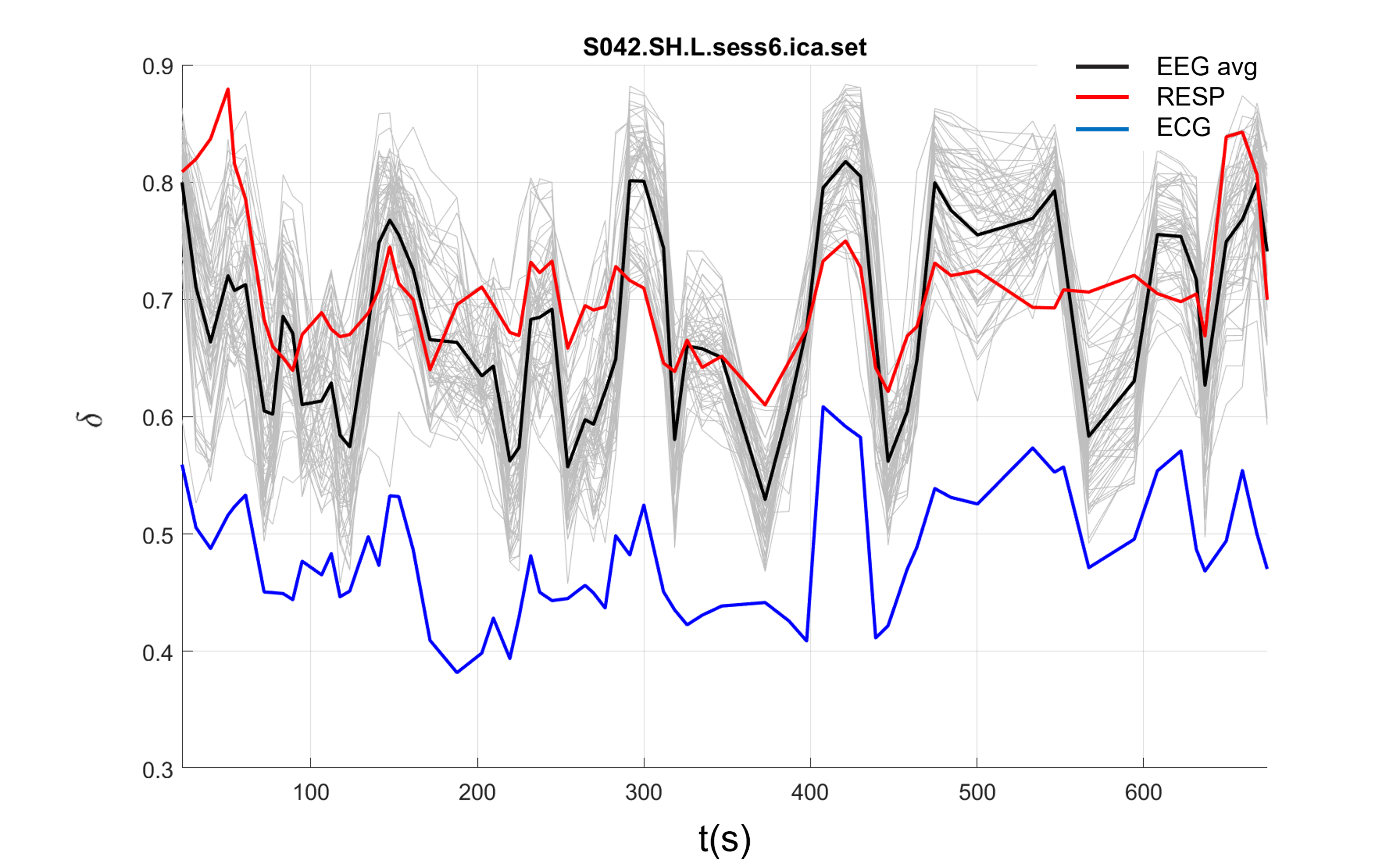

Supplement: Supplementary file 2 [file DataSheet1.zip › Figures/S042.SH.L.sess6.delta_SIG.png]

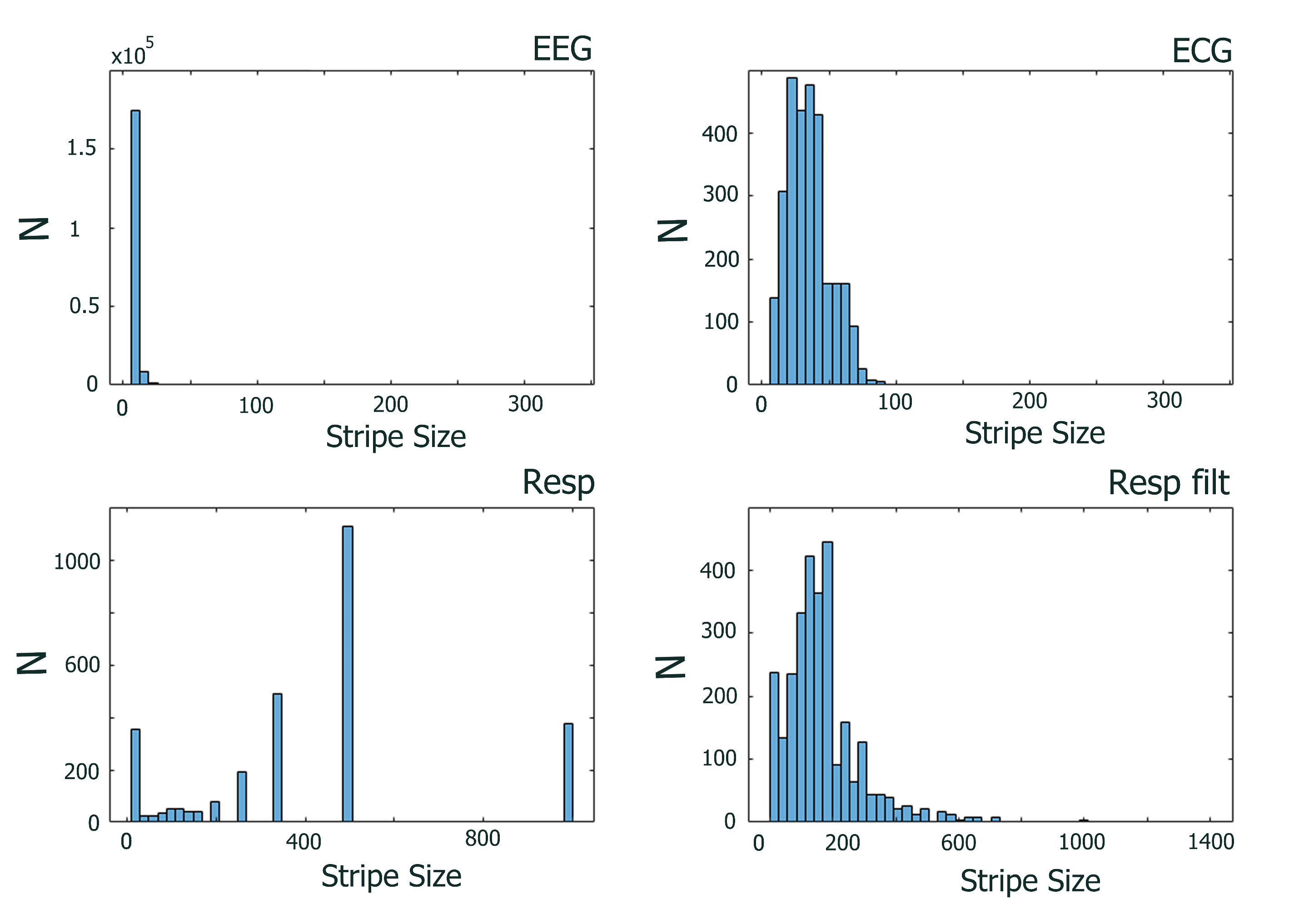

Supplement: Supplementary file 2 [file DataSheet1.zip › Figures/Stripesize_Distributions.png]

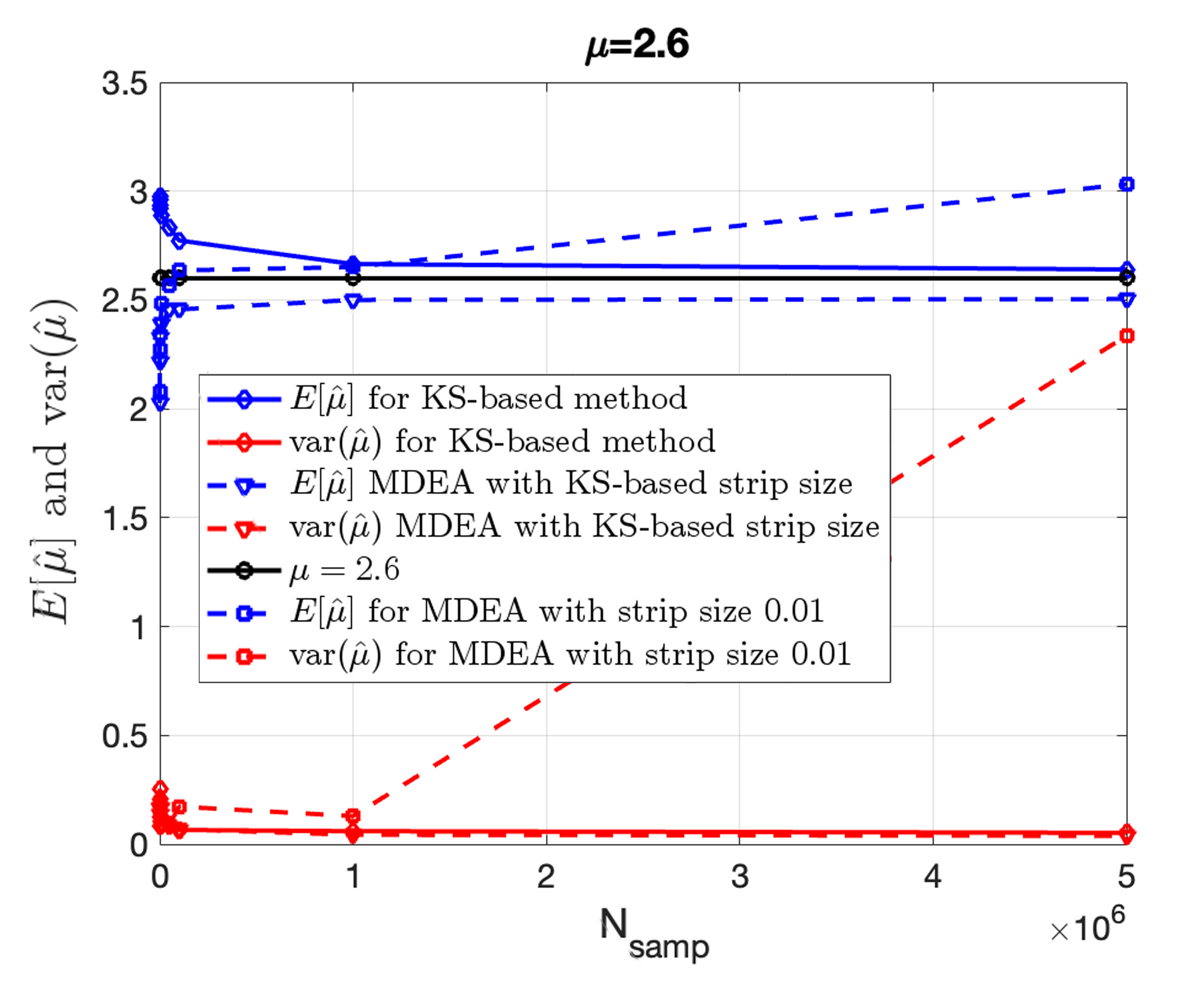

Supplement: Supplementary file 2 [file DataSheet1.zip › Figures/Synth_MittageLeff_mu26.png]

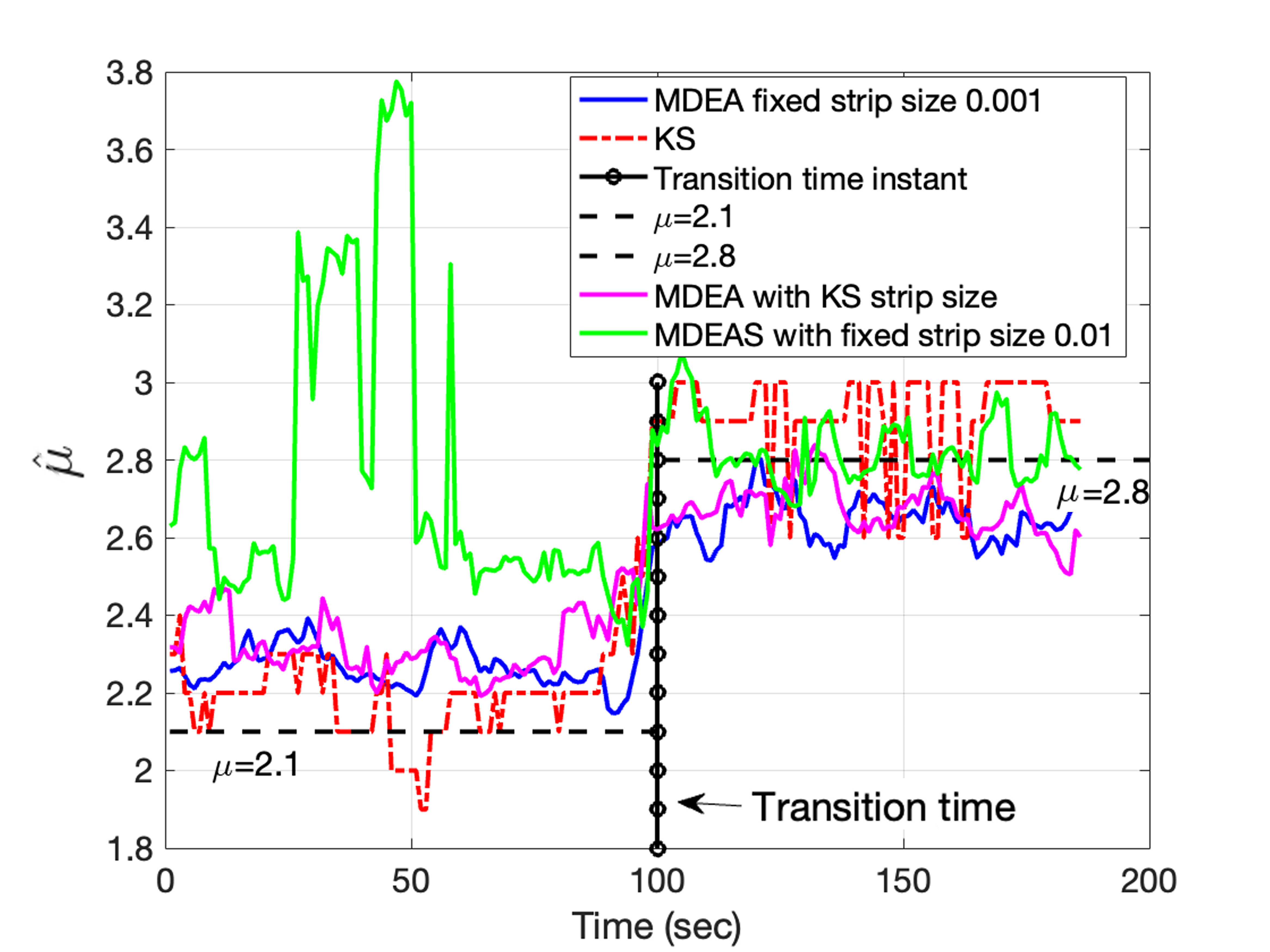

Supplement: Supplementary file 2 [file DataSheet1.zip › Figures/Synth_MittageLeff_TV.png]
